# Cotrimoxazole - optimal dosing in the critically ill

**DOI:** 10.1186/2110-5820-4-13

**Published:** 2014-04-28

**Authors:** Glen R Brown

**Affiliations:** 1 Pharmacy Department, St. Paul’s Hospital, 1081 Burrard St, Vancouver, BC V6Z 1Y6, Canada

**Keywords:** Cotrimoxazole, Trimethoprim, Sulfamethoxazole, Pharmacokinetics, Pharmacodynamics

## Abstract

The optimum dosage regimen for cotrimoxazole in the treatment of life threatening infections due to susceptible organisms encountered in critically ill patients is unclear despite decades of the drug’s use. Therapeutic drug monitoring to determine the appropriate dosing for successful infection eradication is not widely available. The clinician must utilize published pharmacokinetic, pharmacodynamic, and effective inhibitory concentration information to determine potential dosing regimens for individual patients when treating specific pathogens. Using minimum inhibitory concentrations known to successfully block growth for target pathogens, the pharmacokinetics of both trimethoprim and sulfamethoxazole can be utilized to establish empiric dosing regimens for critically ill patients while considering organ of clearance impairment. The author’s recommendations for appropriate dosing regimens are forwarded based on these parameters.

## Review

Cotrimoxazole, the combination of trimethoprim (TMP) and sulfamethoxazole (SMX), is frequently required for the treatment of critically ill patients with infections caused by sensitive pathogens, such as *Pneumocystis jovenii* or *Stenotrophomonas maltophilia*. As with other antibiotics, TMP/SMX must be given in a sufficient dose at a proper frequency to produce adequate concentrations at the site of infection for successful eradication of the pathogen. TMP/SMX also has concentration-dependent toxicities, necessitating avoidance of excessive dosage. The determination of the appropriate dosing regimen requires optimum application of the drug’s pharmacokinetics and pharmacodynamic characteristics. Data from clinical trials of various dosages of TMP/SMX in the critically ill population are generally lacking, forcing the clinician to prescribe the drug without clear knowledge of the appropriate regimen. This paper will review the available pharmacodynamic and pharmacokinetic data necessary for the clinician to determine the optimum dosage of TMP/SMX for selected infections in the critically ill.

Relevant electronic databases of published literature (Embase, Medline) containing studies of the pharmacokinetics, pharmacodynamics, and inhibitory concentrations of TMP/SMX were searched to end date of 16 September 2013. References of selected manuscripts were reviewed for relevant citations. References in tertiary information sources, and the author’s personal information files were searched for relevant data. The preliminary search yielded few studies focused specifically on the critically ill population. Therefore, studies outlining the pharmacokinetics in normal and altered organ clearance populations (renal and/or liver impairment); the pharmacodynamics of TMP/SMX in any setting; and the minimum inhibitory concentrations, determined either *in vitro* or *in vivo*, were selected. Data from the selected publications were reviewed to allow an assessment of the applicability to the critically ill population and to determine potential dosing regimens for specific pathogens in critically ill patients.

### Pharmacokinetics

The volume of distribution (Vd) for each drug has been determined in healthy subjects with TMP having a much larger volume of distribution than SMX based on differences in lipid solubility [1]. There are only very limited data available on the impact of critical illness on the Vd of the two drugs, despite the widely recognized changes that occur with other antibiotics. In critically ill patients requiring mechanical ventilation for *Pneumocystis carinii* pneumonia, the Vd of TMP was 1.6 L/kg versus 1.4 L/kg for non-ventilated patients, and the Vd for SMX was 0.5 L/kg versus 0.4 L/kg [2]. In trauma patients, although reporting low Acute Physiology and Chronic Health Evaluation II (APACHE II) scores of 1 to 24, the Vd of TMP was found to be 2.1 L/kg and for SMX was 0.5 L/kg [3]. No studies have reported the magnitude of change in Vd of the drugs in patients with septic shock requiring large volume resuscitation or vasopressors. Data are also lacking on the impact of obesity on the Vd, and resulting dosing of the drug. Therefore, dosing regimens should be based on actual body weight.

Both TMP and SMX are eliminated from the body predominantly by renal excretion [1,4]. Approximately 20% of SMX is metabolized in the liver to N_4_-acetylsulfamethoxazole which is subsequently excreted in the urine [5]. N_4_-acetylsulfamethoxazole lacks relevant antibacterial activity [1]. The remainder of SMX is cleared by the kidney as unchanged drug [4]. The renal excretion of SMX is increased when the urine is alkaline [5]. Similarly, only a small portion of TMP (10 to 20%) is metabolized by the liver to inactive metabolites, which are subsequently conjugated and excreted in the urine [1]. The remaining portion of TMP elimination is via renal secretion of unchanged drug [4]. Unlike SMX, TMP renal clearance is increased with acid urine [1].

Achievable concentrations for various dosages are described in Table 1. The commercially available tablet contains 80 mg TMP with 400 mg SMX (single strength) or 160 mg TMP with 800 mg SMX (double strength). An intravenous preparation is commercially available containing 16 mg TMP with 80 mg SMX per ml. Both intravenous (IV) and oral (PO) dosages of 15 mg/kg/day of TMP produced Cmax concentrations within the target range for treatment of *Pneumocystis jovenii* (5 to 8 mcg/ml) [2,6]. Oral dosages of 20 mg/kg/day of TMP produced higher concentrations which resulted in a high incidence of toxicities [7]. For pathogens with lower target concentrations (see Target concentrations; Table 2), Cmin concentrations for TMP of above 2 mcg/ml can be maintained with a dosage of 160 mg TMP twice daily.

**Table 1:** Pharmacokinetics of cotrimoxazole (TMP/SMX) in various non-critically ill populations with normal renal and liver function

**Population [Reference]**	**Gender**	**Age (year)**	**Weight (kg)**	**Dose**	**Route**	**Duration**	**Cmax mcg/ml**	**Tmax (min)**	**Cmin mcg/ml**	**Vd (Liters/kg)**	**T1/2 (hours)**	**Clearance (ml/min/kg)**
Normal [8]	1 F/3 M	34	66	80 mg TMP	PO	Q12h × 9 days				1.5	8.2	
				400 mg SMX						0.26	9.8	
Normal [9]	N/A	21 to 40	N/A	240 mg TMP	IV	Q12h × 7 doses	5.91	15	2.64		16.5	
				1,200 mg SMX			178		78		14.1	
Normal [7]	12 M	28	75.8	20.2 mg/kg/day TMP	PO	Q6h × 13 doses	13.6	114		1.4	13.6	1.05
				101.1 mg/kg/day SMX			372	156		0.25	14.0	0.024
Normal [6]	6 M	26.7	73.7	12.6 mg/kg/day TMP	PO	Q6h × 3 days	8.3	90	6.1	1.78	14.6	0.89
				63.3 mg/kg/day SMX			247	108	199	0.27	14.0	0.02
HIV PCP [10]	1 F/22 M	24 to 75	59	15 to 22 mg/kg TMP	IV (15)	Q6h × 4 to 6 days			7.7			
				75 to 110 mg/kg SMX	PO (8)				198			
PCP [2]	N/A	37	67.6	16.1 mg/kg/day TMP	IV	Q6 to 8 h × 2 to 10 days	7.9	15		1.5	11.3	1.73
				80.5 mg/kg/day SMX			186			0.4	14.3	0.34
Ventilated	N/A	37	68.5	14.7 mg/kg/day TMP	IV	Q6 to 8 h × 2 to 10 days	8.1	15		1.6	10.9	1.88
PCP [2]				73.4 mg/kg/day SMX			163			0.5	15.5	0.40
Oncology [11]	N/A	N/A	N/A	150 mg/m^2^ TMP	IV	Q8h × 24 hours	7.02	65	3.65		9.60	
				750 mg/m^2^ SMX			148		88		10.7	
Infected	6 F/5 M	60	65	160 mg TMP	IV	Q8h × 4 days	8.8	60	5.6	0.72	11.3	1.13
Cancer [12]				800 SMX			105.6		70.6	0.27	12.8	0.04
Trauma [3]	2 F/13 M	31	87	8 mg/kg/day TMP	IV	Q12h				2.1	9.8	2.8
				40 mg/kg/day SMX						0.51	8.4	0.75

M, male; F, female; Cmax, maximum concentration within the dosing interval; Tmax, time to reach maximum concentration; Cmin, minimum concentration within dosing interval.

Vd, volume of distribution; T1/2, calculated half-life; N/A, not available; PO, oral administration; IV, intravenous administration; PCP, *Pneumocystis jovenii* pneumonitis.

**Table 2:** Target concentrations for selected pathogens

**Organism**	**Target concentration trimethoprim (mcg/ml)**	**Target concentration sulfamethoxazole (mcg/ml)**	**Reference**
*Pneumocystis jovenii*	5 to 8 Cmax	100 to 200 mcg/ml Cmax	[13]
*Stenotrophomonas maltophilia*	> 6 Cmax	> 60 Cmax	[14]
*Burkholderia pseudomallei*	≥ 2 Cmin	≥ 38 Cmin	[15]
*Nocardia*	≥ 4 Cmin	≥ 76 Cmin	[16]
Methicillin-resistant *Staphylococcus aureus*	≥ 2 Cmin	≥ 38 Cmin	[17]
ESBL Enterobacteriaceae	≥ 2 Cmin	≥ 38 Cmin	[17]

Cmax, Maximum concentration during dosing interval.

Cmin, Minimum concentration during dosing interval.

### Renal dysfunction

Despite the availability of TMP/SMX for a number of decades, published data on the optimum dosage of the drug in patients with renal impairment are unavailable, similar to many widely used treatments [18]. Early work demonstrated a linear relationship between the elimination rate of both unchanged SMX (weak correlation) or TMP (significant correlation) and renal function (as measured by inulin clearance) [8]. The strongest correlation between renal function and pharmacokinetics was seen with the clearance of SMX metabolites (inactive), while the unchanged SMX concentrations remained constant over a wide range of renal impairment [8,19]. These studies involved patients with renal impairment resulting in creatinine clearance rates of 3 to 72 ml/min/1.73 m^2^[8,19]. Using the dosage recommended for normal renal function, similar concentrations of unchanged active SMX from patients with normal renal function were achieved in patients with severe renal impairment [8]. Others, however, have found the elimination of unchanged SMX to be related to renal function, with an estimation of the half-life possible via calculation:

$$T1/2=39\;x\;{\left(\mathrm{CrCl}\;\mathrm{ml}/\min\right)}^{-0.29}\left[20\right]$$

The total amount of SMX (unchanged and metabolites) recovered in the urine of patients with renal impairment remains similar to patients with normal renal function (approximately 80% of an oral dosage), but the portion of the drug remaining unchanged decreased as renal function decreased, suggesting increased metabolism as renal function deteriorates [8]. The magnitude of the increase in clearance can not be estimated clinically or from published literature, but may be of relevance in producing drug concentrations equivalent to patients with normal renal function. The lack of accumulation of active SMX with renal impairment has important therapeutic ramifications since the metabolite does not have antimicrobial activity. If the SMX dosage is reduced when treating patients with renal impairment, the concentration of the active unchanged SMX may decrease, since its clearance is not impaired and may even increase, and potentially compromise the therapeutic effect.

The correlation of renal function and TMP clearance has been verified by other investigators [19,20]. The elimination half-life of TMP has been found to approximate:

$$T1/2=28\;x\;{\left(\mathrm{CrCl}\;\mathrm{ml}/\min\right)}^{-0.13}\left[20\right]$$

Both unchanged drug and the conjugated metabolites of TMP will accumulate as renal function decreases unless a dosage reduction is implemented.

### Liver dysfunction

The pharmacokinetics of both SMX and TMP are not altered significantly by liver impairment [5]. The half-life of both SMX and TMP was not prolonged, versus normal subjects, in patients with mild or severe liver dysfunction, although the characterization of the liver function was poorly defined [21].

The metabolism of SMX appears to be influenced by the activity of N-acetyltransferase 2 (NAT2), with the existence of genetic polymorphism altering the activity of the enzyme [22]. Patients without the native NAT2*4 allele do not clear SMX as rapidly resulting in accumulation of drug and increased exposure [22]. However, the published research methodology does not describe the ability to differentiate active unaltered SMX from the various metabolites. Since they found a correlation between drug accumulation and creatinine clearance, the potential that the finding of increased exposure in patients without native NAT2*4 could be due to accumulation of inactive SMX metabolites. No data exist to describe the effect of critical illness on activity of NAT2 or the ramifications of dosing of patients with the various polymorphisms. Since the individual patients’ genetic makeup is not usually known at the time of initiating TMP/SMX therapy, currently the clinician is unable to assess or consider the need for dosage adjustments.

### Dialysis

Similar to findings in non-dialysis dependent renally impaired patients, unchanged SMX concentrations do not increase in patients with renal impairment sufficient to necessitate dialysis [8]. However, the concentrations of TMP and of SMX metabolites increased in dialysis-dependent patients compared to patients with severe non-dialysis dependent renal impairment [8]. Surprisingly, given the drug’s prolonged use, only limited description of the effect of dialysis on removal of each drug during a dialysis session is available; none for critically ill patients. Pharmacokinetic evaluation in a single patient during a hemodialysis session suggested efficient removal of both antibiotics, with total (unchanged and metabolites) SMX clearance increasing approximately 3.5-fold over non-dialysis periods (83.9 versus 24.4 ml/min) [23]. Approximately 50% of total (unchanged and metabolites) SMX and TMP body stores was removed by a four hour dialysis with a 1 m^2^ cuprophane dialysis membrane at a blood flow rate of 200 ml/min and dialysate flow rate of 500 ml/min [24]. These limited data suggest that hemodialysis is effective in removing SMX metabolites and TMP. However, since active SMX does not accumulate with standard dosing in dialysis patients, the optimum dosing of the combination becomes challenging. Dosage reduction may compromise the activity of unchanged SMX while standard dosages will result in accumulation of potentially toxic SMX metabolites and TMP. A potential approach would be to provide full dosages for the first 24 to 48 hours with subsequent dosage reduction based on clinical response [25].

SMX and its metabolites are partially removed by continuous ambulatory peritoneal dialysis [5], although the amount cleared would be anticipated to be clinically insignificant [23,26]. Instillation of 2 L of 2.5% glucose peritoneal dialysis solution four times daily contributed only 7% of total TMP body clearance and 8% of unchanged SMX clearance in chronic CAPD patients [27]. SMX metabolites are similarly poorly removed by CAPD as demonstrated by a low clearance rate found with four times daily dwells [16]. Rapid cycling peritoneal dialysis at a rate of 3 L/hour efficiently (60%) removed SMX, but had minimal effects on TMP clearance [28]. Hence, peritoneal dialysis should not be considered to reduce the potential for accumulation of either drug to toxic concentrations, and the remaining renal function of the patient should guide dosing.

Removal rates during continuous veno-venous hemodiafiltration (1.8 m^2^ high-flux polysulfone filter at blood flow rates of 180 ml/min, dialysate flow rate of 1,500 ml/hour and fluid replacement of 1,500 ml/hour) produced clearance rates for both drugs similar or exceeding normal renal function [29]. Extended daily dialysis (1.3 m^2^ high flux polysulfone filter at blood flow rates of 140 ml/min, dialysate flow of 170 ml/min for 440 minutes) resulted in substantial clearance of TMP, with less clearance of SMX, and no report of clearance of SMX metabolites [30]. These authors caution clinicians regarding dosing reduction during continuous veno-venous hemodiafiltration and the potential for such reduction to result in inadequate drug concentrations [30].

### Toxic concentrations

The theoretical benefit of utilizing larger dosages of TMP/SMX to ensure the adequacy of achievable concentrations at site of infection must be balanced against the real occurrence of side effects due to excessive drug concentrations (Table 3). Although some TMP/SMX toxicities are predictable, such as the development of hyperkalemia and type 4 renal tubule acidosis from TMP inhibition of sodium channels in the distal nephron, others can not be anticipated. Such unpredictable toxicities are thought to be concentration-related, based on the offending dosages and impaired clearance in many affected patients. However, the concentration required for development of specific toxicities is unknown since the majority of case reports of concentration-dependent toxicities do not have measured serum concentrations at the time of symptoms or signs. However, based on the dosage and the patients’ size and renal function reported in published cases, concentrations required to produce the toxicity would be anticipated to equal or exceed those seen with therapy of 15 mg/kg/day of TMP. This threshold dosage is based on the only clinical trial reporting toxicities with various dosages where dosage regimens producing higher concentrations (Mean for Cmax of TMP 13.6 mcg/ml/SMX 372 mcg/ml) produced an unacceptable incidence of toxicity [7]. Given the large variety of concentration-related toxicities, the clinician can not utilize excessive dosages without exposing the patient to potential risk of toxicity. Similarly, the accumulation of SMX metabolites in renal failure (see Pharmacokinetics above) is anticipated to increase the potential for concentration-related toxicities. However, the balance between effective doses and doses that will produce toxicities with renal impairment is not clear. The truly idiosyncratic toxicities (those that occur at dosages tolerated by the vast majority of patients) cannot be predicted or avoided through dose manipulation.

**Table 3:** Selected cotrimoxazole (TMP/SMX) toxicities and relationship to drug concentration

**Idiosyncratic toxicity**	**Concentration dependent toxicity**
Allergic skin reaction [31]	Apraxia [32]
Cholestasis [33]	Delirium [34]
Hemolytic crisis [35]	Hyperkalemia [36]
Hepatitis [37]	Hypoglycemia [38]
Interstitial nephritis [39]	Aseptic meningitis [40]
Methemoglobinemia [41]	Metabolic acidosis [42]
MODS [43]	Myoclonus [44]
Pancreatitis [45]	Psychosis [46]
Parotitis [47]	Renal tubular obstruction [48]
Stevens-Johnson syndrome [49]	Tremor [50]
Thrombocytopenia [51]	
Torsades de pointe [52]	

MODS, Multi-organ dysfunction syndrome. Square-bracketed numbers indicate references.

### Pharmacodynamics

Both TMP and SMX act by blocking steps in the production of metabolically active folate [53]. Sulfonamides act by blocking the production of dihydrofolate from precursor components (*p*-aminobenzoate, pteridine, and glutamate) [54]. This inhibition is irrelevant for human metabolism since humans can utilize dihydrofolate absorbed from the diet while bacteria must manufacture the dihydrofolate from precursors. TMP works on a subsequent step of folate metabolism by competitively inhibiting dihydrofolate reductase, the enzyme responsible for production of the active tetrahydrofolate [54]. Humans are not adversely affected by the TMP-induced blockage of dihydrofolate reductase since this enzyme in bacteria is greater than 1,000-fold more sensitive than human dihydrofolate reductase. Therefore, at concentrations achieved with therapeutic dosages, two steps in folate metabolism within bacteria are blocked, while human metabolism is unaffected.

Surprisingly, the determination of the pharmacodynamic parameter (concentration or time) most related to bacterial kill has not been clearly determined [55]. TMP/SMX has variable bacteriostatic and bacteriocidal action. Early work suggested that TMP/SMX has bacteriocidal activity against gram positive organisms, such as *Staphylococcus aureus*[56]. What little available evidence evaluating concentration versus time killing suggests that TMP/SMX has concentration-dependent activity against gram-positive organisms, such as *Staphylococcus aureus*[54,57]. For gram-negative organisms, it has been hypothesized that TMP/SMX has concentration-dependent killing of *E. coli*, although supporting data are lacking [57]. Early investigations suggested only bacteriostatic activity with concentrations achievable in urine due to interaction with nutrients which inhibit the activity of the drugs [58]. Subsequent limited evaluation suggests that TMP/SMX is bacteriostatic against sensitive *E. coli* at concentrations of 2 mcg/ml TMP and 40 mcg/ml SMX, supporting time-dependent killing [59]. However, others have shown that at TMP concentrations greater than 2 mcg/ml (>12 mcg/ml SMX) bacteriocidal activity can be detected [20].

For other organisms, the pharmacodynamics are even less clear. Limited evidence from *Burkholderia pseudomallei* treatment suggests concentration effects through achievable *in vivo* concentrations [60]. This concentration-dependent killing was not long standing, and converted to bacteriostatic effects when studied using time-kill methodology [60]. Only bacteriostatic activity could be demonstrated against *Nocardia* spp. at TMP/SMX concentrations of 3.4/17 mg/L; a concentration achievable with a 7.5 mg/kg/day TMP dose) [61].

In summary, TMP/SMX possesses bacteriostatic activity, which is time dependent, with the potential for concentration dependent bacteriocidal activity for specific organisms, possibly in selected infected sites within the body. Since the pharmacodynamics are unclear, clinicians should select dosages that would be anticipated to produce adequate concentrations, as described by successful therapeutic studies, throughout the dosage interval.

### Target concentrations

#### Pneumocystis jovenii

The target concentrations for inhibition of growth of *Pneumocystis jovenii* are thought to span a range of 5 to 8 mcg/ml for TMP and 100 to 200 mcg/ml for SMX at the maximum (Cmax) concentrations of the dosing interval. This range is based on early clinical trials in non-HIV infected patients who had a worse outcome with doses which were unsuccessful in achieving such steady state Cmax concentrations [62-64]. Subsequent trials, again in non-HIV infected patients, demonstrated good clinical recovery when the TMP/SMX dosage was adjusted to achieve a TMP Cmax concentration above 5 mcg/ml [65]. Dosage regimens producing Cmax concentration within a range of 5 to 8 mcg/ml for TMP and 100 to 200 mcg/ml for SMX have been demonstrated to produce more rapid resolution of fever and improvement of alveolar-arterial oxygenation gradient than use of pentamidine [13]. These target concentrations also demonstrated improved survival when compared to use of pentamidine [13]. Dosage regimens producing higher concentrations (mean for Cmax of TMP 13.6 mcg/ml/SMX 372 mcg/ml) have been associated with an unacceptable incidence of toxicity [7]. These data suggest target Cmax concentrations within a range of 5 to 8 mcg/ml for TMP and 100 to 200 mcg/ml for SMX are optimal for *Pneumocystis jovenii* treatment.

#### Stenotrophomonas maltophilia

The target concentrations for eradication of *Stenotrophomonas* infections are unknown. The Clinical and Laboratory Standards Institute (CLSI) standard breakpoint for *Stenotrophomonas* sensitivity to TMP/SMX is < 2 mcg/ml for TMP and 38 mcg/ml for SMX [17]. Approximately 95% of North American *Stenotrophomonas maltophilia* strains were sensitive using the CLSI breakpoint when assessed in the late 1990s [66]. However, other investigators have found that the minimum inhibitory concentration adequate to inhibit 90% (MIC_90_) of isolates is as high as 4/76 mcg/ml [67], a concentration still achievable with oral dosing. Higher concentrations may be desirable based on an *in vitro* model that showed that Cmax concentrations of 6/60 mcg/ml were not bacteriocidal, only bacteriostatic [14]. Very high concentrations (32/608 mcg/ml) are required for bacteriocidal activity, a concentration not achievable or tolerated (see below) [67]. In light of the unlikelihood of achieving bacteriocidal concentrations with tolerable doses, serious *Stenotrophomonas* infections should be treated with alternative antibiotics, such as fluoroquinolones or combinations of betalactam or fluoroquinolones with TMP/SMX [68]. However, infections caused by isolates with lower MICs can be successfully treated, although the optimum serum concentrations have not yet been determined.

#### Burkholderia pseudomallei (Melioidosis)

*Burkholderia pseudomallei* is the causative organism of potentially fatal respiratory infections. The CLSI breakpoints for *B. pseudomallei* are < 2 mcg/ml for TMP and < 38 mcg/ml for SMX [15]. Alternative antibiotics with more favorable MIC and pharmacodynamics, such as cephalosporins or carbapenems, should be considered for acute treatment of serious infections [69]. However, for eradication therapy requiring up to three months of continuous treatment, oral TMP/SMX is an effective and attractive option based on the ease of administering the drug versus parenterally administered alternatives [69]. Based on pharmacodynamic evaluation for *B. pseudomallei*, TMP/SMX was found to be bacteriostatic, so the concentration should be targeted to correspond to the individual isolate MIC [60]. Maintaining the serum concentration above the MIC for 60% of the dosing interval or a 24 hour Area under the concentration curve/MIC ratio of greater than 25 have been suggested as a target pharmacokinetic index [60]. This recommendation has not been tested in a clinical trial evaluating patient outcome. Alternatively, using the dosage of 160/800 mg twice daily for three months has been shown to be effective in eradication of the infection [70].

#### Nocardia spp

*Nocardia* frequently produces life-threatening infections in immunocompromized patients, particularly following solid-organ transplantation. The breakpoint for susceptibility for *Nocardia* spp. is ≤ 2/38 mg/L TMP/SMX [15]. Resistant strains of various *Nocardia* species are frequently encountered [16,71]. The MIC_90_ of many of the resistant strains is 4/76 mg/L [16], a concentration only achievable throughout the dosing interval with high dose TMP/SMX.

#### Methicillin-resistant Staphylococcus aureus (MRSA)

The CLSI breakpoint for methicillin-resistant *Staphylococcus aureus* (MRSA) is identical to that of methicillin-sensitive strains ≤ 2/38 mcg/ml [17]. The prevalence of resistance of MRSA to TMP/SMX varies greatly throughout the world, and may also vary over time within individual institutions, possibly reflecting frequency of TMP/SMX use [72]. If an isolate of MRSA is sensitive to TMP/SMX, the antibiotic is an attractive treatment option. Clinical trials demonstrating clinical efficacy for TMP/SMX in MRSA infections are limited [73]. For an isolate deemed sensitive utilizing the CLSI breakpoint, achievable concentrations with traditional dosing would be expected to be successful in eradicating the pathogen. Small clinical trials have shown such success in treating MRSA bacteremia and other infections, although drug concentrations were not measured [74,75]. However, recent data suggesting that TMP/SMX has negligible effect against intracellular MRSA may limit the clinical utilization of the antibiotic [76].

#### Enterobacteriaceae - extended-spectrum β-lactamase-producing (ESBL) Enterobacteriaceae

TMP/SMX is not altered by beta-lactamases due to the physical structure of each of the antibiotics composing TMP/SMX. However, the majority of organisms which possess the capability to produce ESBLs have also acquired concurrent mechanisms for resistance to TMP/SMX [77]. For example, only approximately 26% of *E. coli* ESBL-producing strains were sensitive to TMP/SMX in a collection of isolates from across Canada in 2007 to 2009 [78]. Exceeding the Standard CSLI breakpoint concentrations (≤2/38) would theoretically be adequate for eradication of sensitive organisms (CSLI). However, evidence supporting the effectiveness or inadequacy of achieving clinical cure when using TMP/SMX for ESBL-producing infections is lacking.

### Dosing recommendations in the critically ill

The clinician is frequently placed in a position of uncertainty regarding the MIC of the infecting organism, and the appropriate dose to achieve the target concentrations for optimal pharmacodynamics, and resulting efficacy. Clinical trials to demonstrate the optimal dosing regimens, as discussed above, are generally lacking. The benefit of prospective therapeutic drug monitoring of TMP/SMX concentrations in critically ill patients has not been evaluated in the published literature. Intuitively, therapeutic drug monitoring would be anticipated to be beneficial. However, the capability of most clinical laboratories to provide such a service is limited. Therefore, the clinician is forced to extrapolate the available pharmacodynamic and pharmacokinetic information available to select a dosage regimen. Due to the lack of published literature on the effects of critical illness and treatment on the pharmacokinetics of TMP/SMX, the clinician must attempt to estimate dosing requirements. Similarly, the lack of data on the impact of obesity on dosing requirements forces the clinician to base dosing on actual body weight to ensure achievement of desired drug concentrations. The urgency of obtaining effective concentrations in a patient who is critically ill from an infection warrants the use of doses larger than in patients with limited symptoms from the infection. By utilizing the limited available data on pharmacokinetics, pharmacodynamics, and potential for dose-related toxicities, the following doses are recommended for achieving the desired target concentrations for potential pathogens in critically ill patients.

For patients with *Pneumocystis jovenii*, *Stenotrophomonas maltophilia, Burkholderia pseudomallei or Nocardia* spp. infection - 15 mg/kg/day TMP IV or PO (with corresponding 75 mg/kg/day SMX).

(The drug could be given in two divided dosages based on the pharmacokinetics, but the relationship between Cmax concentrations and toxicity has been only evaluated in a limited non-critically ill population [7]. Whether the toxicity is more closely correlated with the Cmax or total exposure to the drugs is not known. To potentially minimize the impact of the Cmax on development of toxicities, the drug can be administered in smaller doses more frequently, three to four per day.)

For methicillin-resistant *Staphylococcus aureus* (MRSA) or extended-spectrum β-lactamase-producing (ESBL) Enterobacteriaceae - 160 mg TMP IV or PO twice daily (with corresponding 800 mg SMX).

For patient with renal impairment:

a) Measured or estimated creatinine clearance:

Greater than 30 ml/min - full dose;

Fifteen to 30 ml/min - half dose (the concentration of active SMX may be reduced, relying on TMP activity only);

Less than 15 ml/min - avoid drug if possible since the SMX metabolites will accumulate to potentially toxic concentrations unless dosage reduction in implemented, which would result in inadequate active SMX exposure.

b) Hemodialysis-dependency - 7.5 mg/kg after each four hour hemodialysis session (initial 24 to 48 hours at 15 mg/kg/day).

c) Continuous Ambulatory Peritoneal Dialysis - avoid drug if possible since the SMX metabolites will accumulate to potentially toxic concentrations unless dosage reduction in implemented, which would result in inadequate active SMX exposure.

d) Continuous Veno-venous Hemodiafiltration or Slow extended daily hemodialysis - 15 mg/kg/day TMP for *Pneumocystis jovenii* and equivalent infections; avoid underdosing with CVVHDF to avoid inadequate concentrations.

e) Liver Failure - no dosing adjustment needed.

## Conclusions

Limited documentation of appropriate concentrations for treatment of critically ill patients forces the clinician to use available pharmacodynamic, toxicologic, and pharmacokinetics of both TMP and SMX in determining dosing regimens for critically ill patients considering organ of clearance impairment.

## Abbreviations

APACHE II: Acute Physiology and Chronic Health Evaluation II; CAPD: Continuous Ambulatory Peritoneal Dialysis; CLSI: Clinical and Laboratory Standards Institute; Cmax: maximum concentration within the dosing interval; Cmin: minimum concentration within dosing interval; CrCl: creatinine clearance; CVVHDF: Continuous Veno-venous Hemodiafiltration; ESBL: extended-spectrum β-lactamase-producing; IV: intravenous; MIC90: minimum inhibitory concentration adequate to inhibit 90%; MRSA: methicillin-resistant *Staphylococcus aureus*; NAT2: N-acetyltransferase 2; PCP: *Pneumocystis* pneumonitis; PO: oral; SMX: sulfamethoxazole; T1/2: half-life; Tmax: time to reach maximum concentration; TMP: trimethoprim; Vd: volume of distribution.

## Competing interests

The author declares no financial, professional, political, personal, religious, ideological, academic, intellectual, commercial or other relationship that would be a conflict of interest in the interpretation of any information related to TMP/SMX.

## Authors’ contributions

The sole author is responsible for all aspects of preparation, writing, and submission of this manuscript.

## Authors’ information

The author is a clinical pharmacist at St. Paul’s Hospital in Vancouver, British Columbia where he has practiced for 25 years. The author is a Clinical Professor, Faculty of Pharmaceutical Sciences, University of British Columbia.
